# 2375 Pharmacokinetics of phosphatidylethanol 16:0/20:4 homolog in human blood after consumption of 0.4 and 0.8 g/kg alcohol in a laboratory clinical study

**DOI:** 10.1017/cts.2018.107

**Published:** 2018-11-21

**Authors:** Marisa Lopez-Cruzan, Nathalie S. Hill-Kapturczak, Jesus J. Sanchez, John D. Roache, Tara Wright, Donald Dougherty, Martin Javors

**Affiliations:** University of Texas Health Science Center San Antonio

## Abstract

OBJECTIVES/SPECIFIC AIMS: The purpose of this study was to characterize the pharmacokinetics of phosphatidylethanol (PEth) 16:0/20:4 homolog in uncoagulated, human blood samples taken from 18 participants in a clinical laboratory setting after consumption of 2 doses of ethanol. METHODS/STUDY POPULATION: Male and female participants received either 0.4 or 0.8 g/kg oral doses of ethanol during a 15-minute period. Blood samples were collected before and throughout 6 hours immediately after alcohol administration, then after 2, 4, 7, 11, and 14 days of administration day. PEth 16:0/20:4 levels were quantified by liquid mass spectrometry. Breath ethanol concentrations were measure concurrently with each blood collection during the administration day, as well as transdermal ethanol concentrations monitored constantly before, during and after ethanol administration day. RESULTS/ANTICIPATED RESULTS: (1) Single doses of 0.4 and 0.8 g ethanol/kg produced proportional increases in BrAC and PEth 16:0/20:4 levels; (2) the increase of Peth 16:0/20:4 from base line to Cmax was less than either PEth 16:0/18:1 or PEth 16:0/18:2 during the 6-hour period after ethanol administration; (3) the mean rate of formation of PEth 16:0/20:4 was lower than those of the other 2 homologs; (4) the mean half-life of PEth 16:0/20:4 was 2.18 days, which was shorter than that of either PEth 16:0/18:1 and PEth 16:0/18:2, which were 6.80 and 6.62, respectively. DISCUSSION/SIGNIFICANCE OF IMPACT: The results of this study further confirm that PEth homologs are a sensitive biomarker for ethanol consumption. The measurement of three PEth homologs appears to provide additional information about the level and time frame of drinking.

